# Macrophage-like THP-1 cells show effective uptake of silica nanoparticles carrying inactivated diphtheria toxoid for vaccination

**DOI:** 10.1007/s11051-019-4720-1

**Published:** 2020-01-08

**Authors:** Xinyue Huang, Danielle Paixão Cavalcante, Helen E Townley

**Affiliations:** 1 Nuffield Department of women’s and reproductive health, Oxford University, John Radcliffe Hospital, Oxford, UK; 2 Cristália Produtos Químicos Farmacêuticos Ltda, Sao Paulo, Brazil; 3 Department of Engineering Science, Oxford University, Park’s Road, Oxford, UK

**Keywords:** Nanoparticle, Vaccine, Antigen, Silica, Macrophage, Nanomedicine

## Abstract

Nanoparticles may be used in vaccinology as an antigen delivery and/or an immunostimulant to enhance immunity. Porous silica has been identified as an effective adjuvant for more than a decade, and we have therefore investigated the take up rate by an immortalized macrophage-like cell line of a number of mesoporous silica nanoparticles (MSNPs) with differing diameter and pore size. The MSNPs were synthesized using a sol-gel reaction and post-synthesis removal of the template. The MSNPs showed a clear distribution in take up rate peaking at 217 nm, whereas a comparison with solid spherical nanoparticles showed a similar distribution peaking at 377 nm. The MSNPs were investigated before and after loading with antigen. Diphtheria toxoid was used as a proof-of-concept antigen and showed a peak macrophage internalization of 53.42% for loaded LP3 particles which had a diameter of 217.75 ± 5.44 nm and large 16.5 nm pores. Optimal MSNP sizes appeared to be in the 200–400 nm range, and larger pores showed better antigen loading. The mesoporous silica particles were shown to be generally biocompatible, and cell viability was not altered by the loading of particles with or without antigen.

**Figure:**
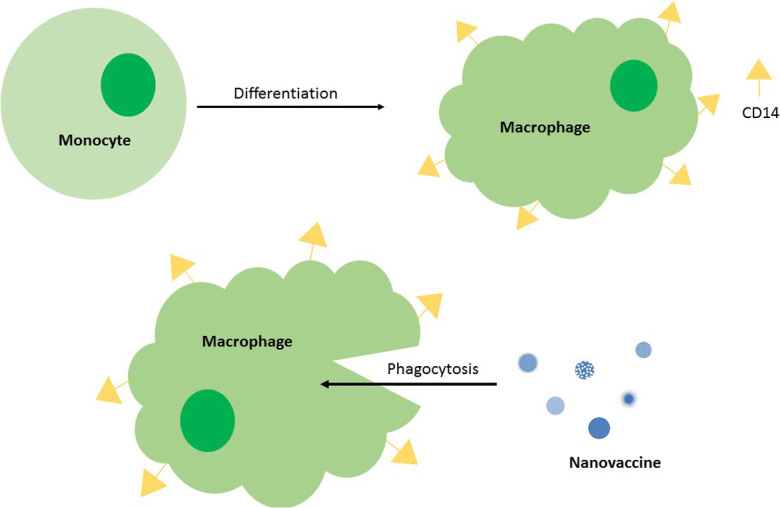
Graphical abstract

Graphical abstract

## Introduction

Nanovaccinology involves the use of nanometre sized particles as a means to deliver antigen, in order to enhance antigen processing. A variety of engineered nanoparticles have been developed for vaccine development and while they differ in size, shape and composition, they are typically 25–200 nm in diameter (Zhao et al. 2014). The large surface area to volume ratio of nanoparticles means that the particles may carry a higher proportion of antigen for immune activation. The size of the nanoparticle systems also increases the likelihood that they will accumulate in lymph nodes. This can both increase their effective concentration and reduce systemic reactivity (Nuhn et al. 2016). Our previous work compared the antigen loading and unloading capacity of mesoporous silica nanoparticles (MSNPs), with a variety of different pore sizes, and external diameters (Huang et al. submitted). Herein, we investigate the ability of the different particles to be internalized by antigen-presenting cells. The uptake of nanoparticles, and subsequent immune response, may be due to their intrinsic resemblance to natural viruses (Chattopadhyay et al. 2017). Since the immune system is primed to recognize viruses, it is not surprising that nanoparticles which can be similar in terms of size, geometry and antigen display could be effective. Soluble antigens are well known to have low efficacy in inducing good protective immunity. This is largely due to the insufficient uptake of the antigen by antigen-presenting cells (APCs). Attaching soluble vaccine antigens to larger carriers can facilitate recognition and uptake by the APCs (Pobre et al. 2014).

Materials used for the production of nanoparticles for antigen delivery include lipids, polymers and inorganic compounds. For our study, we have chosen to investigate the use of silica nanoparticles as a carrier for antigen molecules due to a number of favourable properties: Silica nanoparticles can be synthesized in a range of sizes and shapes and can be controlled using sol-gel chemistry and is a particularly useful material to work with since nanoscale pores can be easily integrated to generate MSNPs which have a very large surface area for carrying antigen. In addition to the size of the particles, charge is also an important parameter. Some studies have shown that cationic nanoparticles show higher uptake due to the interaction with anionic cell membranes (Foged et al. 2005). However, cationic particles are more likely to induce haemolysis and platelet aggregation than neutral or anionic particles (McCusker et al. 2004). The negatively charged silanol groups found on the surface of silica are also beneficial if further modification with additional functional molecules such as those for cell recognition is needed.

The attachment of a vaccine antigen to a nanoparticle may be via chemical conjugation or physical adsorption. In this study, we have used physical adsorption which is simply based on the charge or hydrophobic interaction of the antigen and the nanoparticle material. Conjugation could potentially result in modification of the antigen as a result of the binding to the nanoparticle surface, potentially leading to a less immunogenic antigen.

In addition, silica has been shown to be biocompatible, and the pore size can be carefully controlled to determine the optimal size to adsorb and deliver antigen. While the silica itself can be used as an adjuvant and is capable of inducing strong humoral and cellular immune responses against the relevant antigens, others can also be added as cargo (Mahony et al. 2013). To date, silica has been investigated in formulations for vaccine applications against *E. coli*, porcine circovirus and HIV (Guo et al. 2012; Mercuri et al. 2006; Cheng et al. 2012). Furthermore, in contrast to other nanoparticles such as gold, silica is also easily degraded (Huang et al. 2014) in the body and can then be excreted in the urine.

Irrespective of the material chosen, to be effective as a vaccine delivery system, it is essential that the micro- or nanoparticles are internalized by antigen-presenting cells (Alshanqiti et al. 2017). We have therefore investigated the uptake of our library of silica nanoparticles using the human cell line THP-1 as a model system. THP-1 is an immortalized monocyte-like cell line, derived from the peripheral blood of a childhood case of monocytic leukaemia (M5 subtype) (Tsuchiya et al. 1980). These cells can be differentiated into macrophage-like cells using phorbol-12-myristate-13-acetate (PMA) which activates protein kinase C (PKC). The resulting cells resemble mature macrophages, with increased adherence and loss of proliferative activity (Schwende et al. 1996). The cells can be confirmed to be macrophage-like by the expression of CD-14, a 53–55 kDa glycosylphosphatidylinositol (GPI)-linked membrane glycoprotein.

As a proof-of-concept, we have loaded the nanoparticles with the inactivated diphtheria toxoid (DT). DT is secreted as a single polypeptide of 58 kDa by pathogenic strains of *Corynebacterium diphtheria*. Subsequently, the 535 amino acid polypeptide is proteolyzed to two polypeptide chains linked by a disulphide bond. The DT is well characterized and has been used since the 1920s when the first methods to inactivate the toxoids were introduced (Ramon 1924; Glenny and Hopkins 1923). This study therefore investigates the uptake rates of macrophages of loaded and unloaded MSNPs, with different external diameters and pore sizes.

## Materials and methods

### THP-1 cell culture

THP-1 cells were donated by Dr. Paul Klenerman (Nuffield Department of Medicine, University of Oxford). Cells were maintained in RPMI 1640 media supplemented (Life Technologies) with 10% fetal calf serum (FCS; Sigma-Aldrich), 2 mM L-glutamine (Sigma-Aldrich), 100 U/mL penicillin (Sigma-Aldrich), 0.1 mg/mL streptomycin (Sigma-Aldrich) and 0.05 mM 2-mercaptoethanol (Sigma-Aldrich). Cells were incubated at 37 °C in a 5% CO_2_ atmosphere. Cells were passaged every 4–5 days and discarded after 30 passages.

### THP-1 cell differentiation

THP-1 cells (from 5th generation to 30th) were resuspended in RPMI media at a concentration of 2 × 10^5^ cells/mL and treated with 100 ng/ mL phorbol 12-myristate 13-acetate (PMA; Sigma). Cells were then plated in a 24-well plate (1 mL per well) and incubated for 72 h at 37 °C, 5% CO_2_. The PMA-containing media was then removed and replaced with fresh RPMI media for 24 h before further experiment.

### Evaluation of differentiation

The CD-14 marker on PMA-differentiated and undifferentiated cells were compared by flow cytometry since CD-14 is considered a surface marker of macrophages. THP-1 cells were collected and washed with PBS. The Fc receptor was blocked by resuspending cells at 1 × 10^4^ cells/ mL in 5 μg/ mL IgG (Sigma) in PBS at a total volume of 100 μL and incubating on ice for 20 min. Subsequently, 5 μL of APC-CD-14 primary antibody (BioLegend) was added and incubated on ice for a further 20 min. Cells were collected by centrifugation and the supernatant removed. Cells were washed in 200 μL PBS and then centrifuged and resuspended in 200 μL PBS.

Controls were also prepared, including undifferentiated THP-1 cells incubated with only IgG and undifferentiated cells incubated with both IgG and anti-CD14 antibodies.

### Phagocytosis assay

The differentiated THP-1 cells (2 × 10^5^ cell/well) were treated with 10 μg/mL fluorescein isothiocyanate (FITC)-labelled MSNPs for 2 h at 37 °C. After the treatment, the cells were resuspended and fixed in 100 μL cold 2% paraformaldehyde for 10 min on ice. The phagocytosis efficacy was evaluated using FACS.

The negative control was performed on low temperature (4 °C) while the other parameters of MSNP treatment remain the same. The net phagocytosis efficacy was evaluated by subtracting the result of the negative control for the experiment.

### Fluorescence activated cell sorting

Fluorescence activated cell sorting (FACS) was run on a FACSCalibur™. All data was collected using CellQuest™ Pro (BD Biosciences). The data was analysed using Kaluza™ 1.2.

### Viability assay

Cells were incubated with nanoparticles for 2 h. The particle suspension was then removed and replaced with 100 μl of 0.5 mg/mL MTT (3-(4,5-dimethylthiazol-2-yl)-2,5-diphenyltetrazolium bromide; Life Technologies) solution in culture medium. The plate was wrapped in foil and the cells were incubated for a further 2 h, at 37 °C. An aliquot of the media (75 μl) was removed and 150 μl of dimethyl sulfoxide (DMSO; Sigma) was added to the plate. The absorbance was read at 590 nm after solubilization of the precipitate.

### Synthesis protocol for solid silica nanoparticles

#### Synthesis of SNP01, 02, 03

Absolute ethanol (48 mL; Fisher Scientific) was mixed with 3 mL deionized water and 3 mL ammonium hydroxide (28–30%; Sigma) in a flask and placed on a heated stirrer. The temperature was either set to (i) 75 °C for SNP01, (ii) 48 °C for SNP02 or (iii) 52 °C for SNP03. Once the temperature was stable, 3 mL tetraethyl orthosilicate (TEOS; Sigma-Aldrich) was added. The mixture was then stirred at the same temperature overnight. The particles were subsequently collected by centrifugation and washed three times in ethanol. The particles were then dried in a desiccator and ground using a mortar and pestle.

#### Synthesis of SNP04 and SNP05

Absolute ethanol (30 mL) was mixed with 3 mL of 0.25 mg/mL potassium chloride solution. Ammonium hydroxide (28–30%) was added at either (i) 1.5 mL for SNP04 or (ii) 3 mL for SNP05, and the mixture placed on a heated stirrer at 35 °C. In a separate tube, 3.5 mL TEOS was mixed with (i) 21 mL ethanol for SNP04 or (ii) 10.5 mL ethanol for SNP05 and vortexed (mix 2). Once the temperature had stabilized, either (i) 21 mL for SNP04 or (ii) 12 mL for SNP05, of mix 2 was added to the first flask. The final mixture was incubated at 35 °C for either 4 h (SNP04) or overnight (SNP05). The particles were subsequently collected by centrifugation and washed three times in ethanol. The particles were then dried in a desiccator and ground using a mortar and pestle.

### Synthesis protocol of MSNPs

Seven different mesoporous silica nanoparticles (MSNPs) were synthesized with varying external diameter and porosity. The first four had small pores (SP) and were designated SP1, SP2, SP3 and SP4. The latter three had much larger pores (LP) and were named LP1, LP2 and LP3. Santa Barbara Amorphous particles (SBA-15) were synthesized by Cristália Produtos Químicos Farmacêuticos Ltda, Brazil. These were included as a comparison since they have been used previously in adjuvant studies in the literature.

### Synthesis protocol of SP1

Particles were made after the method of Zukal et al. (2007). In a typical synthesis process, 300 mg of 1-methyl-3-octylimidazolium chloride (OMIMCl), 100 mg of cetyl trimethylammonium bromide (CTAB) and 400 mg of sodium silicate (Na_2_SiO_3_) were dissolved in 90 mL of ddH_2_O in a 250 mL round-bottom glass flask at room temperature. After the solution cleared by stirring for at least 1 h, 0.5 ml of ethyl acetate was quickly added whilst stirring. After 1 min, the stirring was stopped, and within 10 min at room temperature, a precipitate began to form. The mixture was allowed to stand at 35 °C for a further 5 h. The suspension was then heated to 95 °C, and the mixture stirred slowly for 48 h. During the ageing process, organic vapour was allowed to escape. The resulting solid phase was recovered by filtration, washed three times with 30 mL ddH_2_O, and then washed a further three times with 30 mL MeOH. The template was removed as below.

### Template removal

To remove the surfactant, the particles were resuspended in acidic methanol (40 mL methanol; 2 mL 37% hydrochloric acid). The system was then refluxed at 80 °C for 24 h. After reflux, the suspension was allowed to cool down to room temperature, and particles collected and washed as in “THP-1 cell culture”, with the exception that ethanol was used for washing in place of methanol. The particles were dried in a desiccator under high vacuum for at least 24 h at room temperature. Subsequently, the particles were ground to a fine powder using a mortar and pestle.

### Synthesis protocol of SP2

Particles were made after the method described in Huang et al. (2014). In a typical synthesis process, 200 mg (CTAB) was dissolved in 96 mL ddH_2_O, and 700 μL of 2 M sodium hydroxide (NaOH) solution was added, in a 250-mL round-bottom glass flask. The mixture was then stirred and heated to 80 °C. Once the temperature was attained, 1 mL tetraethyl orthosilicate (TEOS) was added, and the reaction kept at 80 °C for 2 h. The mixture was then allowed to cool to room temperature and the particles collected by centrifugation for 3 min at 12,000 rpm for 3 min. The particles were then resuspended in 30 mL MeOH and centrifuged. This washing step was repeated three times.

### Synthesis protocol of SP3

Particles were made after the method of He et al. (2009). In a typical process, 365 mg CTAB was dissolved in 99.5 mL buffer solution (comprising 25 mM KH_2_PO_4;_ 14.5 mM NaOH; pH 7) and 0.5 mL glycerol under vigorous stirring at 95 °C. When the solution became homogeneous, 1.78 mL TEOS was added slowly into the system. The reaction was maintained at 95 °C for 8 h, washed three times with 30 mL ddH_2_O, and then washed a further three times with 30 mL MeOH. The template was removed as described above.

### Synthesis protocol of SP4

Particles were made after the method of Du and He 2010. In a typical synthesis process, 800 mg CTAB was dissolved in a mixture of 100 mL ddH_2_O, 0.8 mL NH_4_OH, 20 mL ether and 20 mL ethanol, with constant stirring in a 250-mL round-bottom glass flask at room temperature. After a clear solution was obtained, 2.5 mL TEOS was added to the solution. After vigorous stirring for 4 h at room temperature, a white precipitate was obtained. The particles were collected by centrifugation and resuspended in 30 mL methanol and centrifuged again. This washing step was repeated twice more. The template was removed as described above.

### Synthesis protocol of LP1

In a typical synthesis, 1 g CTAB was added to 15 mL 2 M urea solution and stirred vigorously at room temperature. To the mixture, cyclohexane (25.6 mL) and butan-1-ol (1.234 mL) were added and the system heated at 40 °C for at least 1 h before proceeding. TEOS (2.68 mL) was then added dropwise and stirring continued for 30 min before increasing the heat to 70 °C for another 24 h. The system was then cooled to room temperature, and 100 mL ethanol added. The particles were then collected by centrifugation at 12,000 rpm for 5 min. The particles were then resuspended in 30 mL acetone and centrifuged again. This wash step was repeated once, and then repeated twice using water, and then repeated twice using ethanol to wash. The template was removed as described above.

### Synthesis protocol of LP2

In a typical synthesis, 500 mg cetylpyridinium bromide (CPB) was dissolved in 15 mL urea (20 mg/mL) and stirred vigorously at room temperature. Cyclohexane (15 mL) was added while stirring, followed by 0.55 mL butan-1-ol. The solution was stirred for 3 h. Subsequently, 1.34 mL of tetraethyl orthosilicate (TEOS) was added dropwise into the system and stirring continued for another 30 min at room temperature. The reaction mix was then maintained at 70 °C for a further 16 h. After removal from the heat, 100 mL ethanol was added. The nanoparticles were then collected by centrifugation at 12,000 rpm for 5 min. The particles were then washed with acetone and re-centrifuged, followed by resuspension in water and centrifugation. The template was removed as described above.

### Synthesis protocol of LP3

The protocol was as for LP2 with the exception that butan-1-ol was replaced with 0.65 mL pentanol.

### Synthesis protocol of SNP01

Absolute ethanol (48 mL) was added to deionized water (3 mL) and ammonium hydroxide (3 mL; 28% to 30%) and the mixture heated to 55 °C. TEOS (3 mL) was added to the system, and the mixture was stirred at 55 °C for 16 h. The particles were collected by centrifugation and washed three times with ethanol. The particles were dried in a desiccator and ground using a mortar and pestle.

### FITC labelling of particles

FITC-APTES was prepared in an anaerobic chamber by the addition of 100 μl of 3-aminopropyl triethoxysilane (APTES; Sigma) to 25 mg fluorescein isothiocyanate (FITC; Sigma) in 5 mL dry ethanol. The mixture was covered to protect from light and stirred overnight at room temperature. The resulting FITC-APTES was stored at 4 °C.

For labelling, silica nanoparticles were resuspended at 25 mg/mL in ethanol. The suspension was sonicated using a probe sonicator for 2 min, and then 20 μl of FITC-APTES was added to every millilitre of nanoparticle suspension and stirred overnight at room temperature. The labelled nanoparticles were collected by centrifugation, and then washed three times with ethanol. The particles were dried in a desiccator with care to avoid exposure to light.

### Zeta potential

The surface charge of the particles was measured using a Zetasizer Nano ZS (Malvern, UK). To determine the electrokinetic potential, or ζ potential, the particles were suspended in distilled water (pH 7) prior to measurement. The suspension was used to fill a DTS1070 disposable capillary cell. After 120 s equilibrium time, 30 runs were read before the calculation of zeta potential.

### Disc centrifuge

The hydrodynamic particle size distributions were determined using a Disc Centrifuge (DC24000; CPs instrument). Prior to measurements, a sucrose gradient was built and PVC particle calibration standards were applied (266 nm; PVC000266, Analytic Ltd.).

### Antigen loading of nanoparticles

The diphtheria toxoid was kindly provided by Fundação Butantan to Cristália Produtos Químicos Farmacêuticos Ltda. Silica nanoparticles were resuspended in PBS at a concentration of 5 mg/mL. The MSNP stock suspension was sonicated using a VibraCell VC500 sonicator for 1 min (5 s on/ 5 s off). Inactivated diphtheria toxoid (DT) added to a final concentration of 2 mg/mL, and mixed well. Loading was allowed to proceed for 24 h at 4 °C.

### Statistical analysis

All data is presented as the mean ± standard deviation, and where appropriate, the student *t* test was used to determine statistical significance (*p < 0.05, **p < 0.01, ***p < 0.005).

## Results

**Fig. 1:**
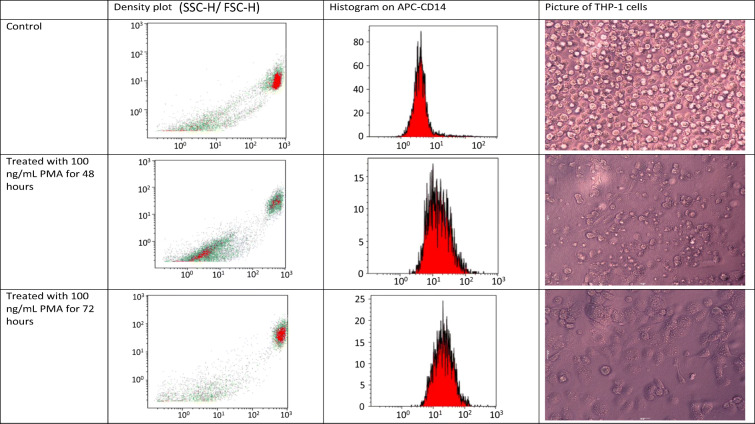
Differentiation of THP-1 cells after incubation with PMA **a** Control; no PMA, **b** after treatment with 100 ng/mL PMA for 48 h, **c** after treatment with 100 ng/mL PMA for 72 h. Panel (i) shows light scatter plot of cells. Panel (ii) FL3 (red channel to detect APC-CD-14) indicates the expression of CD14 on THP-1 cells. Panel (iii) Microscope images show the morphology of the cells

To assess the uptake of the different silica nanoparticles, and to determine whether this was affected by size, porosity or loading, we tested the ability of the particles to be taken up into macrophage-like cells. In all phagocytosis assays performed, THP-1 cells were used and differentiated using 100 ng/mL PMA for 72 h. The light scatter plot (Fig. 1; panel (i)) shows the changes in the populations of cells before and after treatment with PMA to differentiate the cells. The population of cells in the control and after treatment with PMA for 72 h shows homogeneous cell populations, whereas treatment for 48 h is an intermediate state with two populations of cells. To confirm the differentiation of the THP-1 cells, CD-14 expression (a surface marker normally overexpressed in macrophages) was evaluated. An APC-labelled CD-14 antibody shows emission in the 660 nm (red) region and was used to assess the expression of the glycoprotein (Fig. 1; panel (ii)). The median fluorescence intensity increases from 3.95 (control) to 14.73 and 20.98 after incubation with PMA for 48 and 72 h, respectively. Therefore, PMA can be seen to have successfully increased the expression of CD-14 and by implication resulted in the differentiation of the cells into macrophages. As further confirmation, the cells were examined microscopically (Fig. 1; panel (iii)). The morphology of the cells can be seen to change over the period of incubation with PMA and to give the appearance of macrophages after 72 h.

Solid, spherical nanoparticles were synthesized in a range from 113.25 ± 7.34 to 847.25 ± 27.54 nm in diameter (Table 1). The nanoparticles were imaged by SEM to confirm morphology and the diameter assessed using CPS disc centrifuge. Multiple batches were prepared for each to ensure that the methodology was robust and repeatable.

**Table 1:** The diameter of the solid spherical nanoparticles was assessed using CPS disc centrifugation. Zeta potential was determined using a Zetasizer Nano ZS (Malvern UK). Number of replicates indicates number of different batches of particles made and assessed. SBA-15 was assessed by SEM (due to rod-like shape). Number of replicates relates to the number of particles measured in the sample

Name	Diameter (nm)	Potential (mV)
SNP01	113.25 ± 7.34 (*n* = 8)	− 19.93 ± 3.36
SNP02	275.78 ± 17.75 (*n* = 9)	− 27.27 ± 1.00
SNP03	377.63 ± 11.36 (*n* = 8)	− 45.17 ± 1.33
SNP04	588.00 ± 25.61 (*n* = 7)	− 45.20 ± 0.44
SNP05	847.25 ± 27.54 (*n* = 8)	− 50.70 ± 0.62
	Length (nm)	
SBA15	612.75 ± 82.22 (*n* = 13)	− 19.70 ± 0.30

**Fig. 2:**
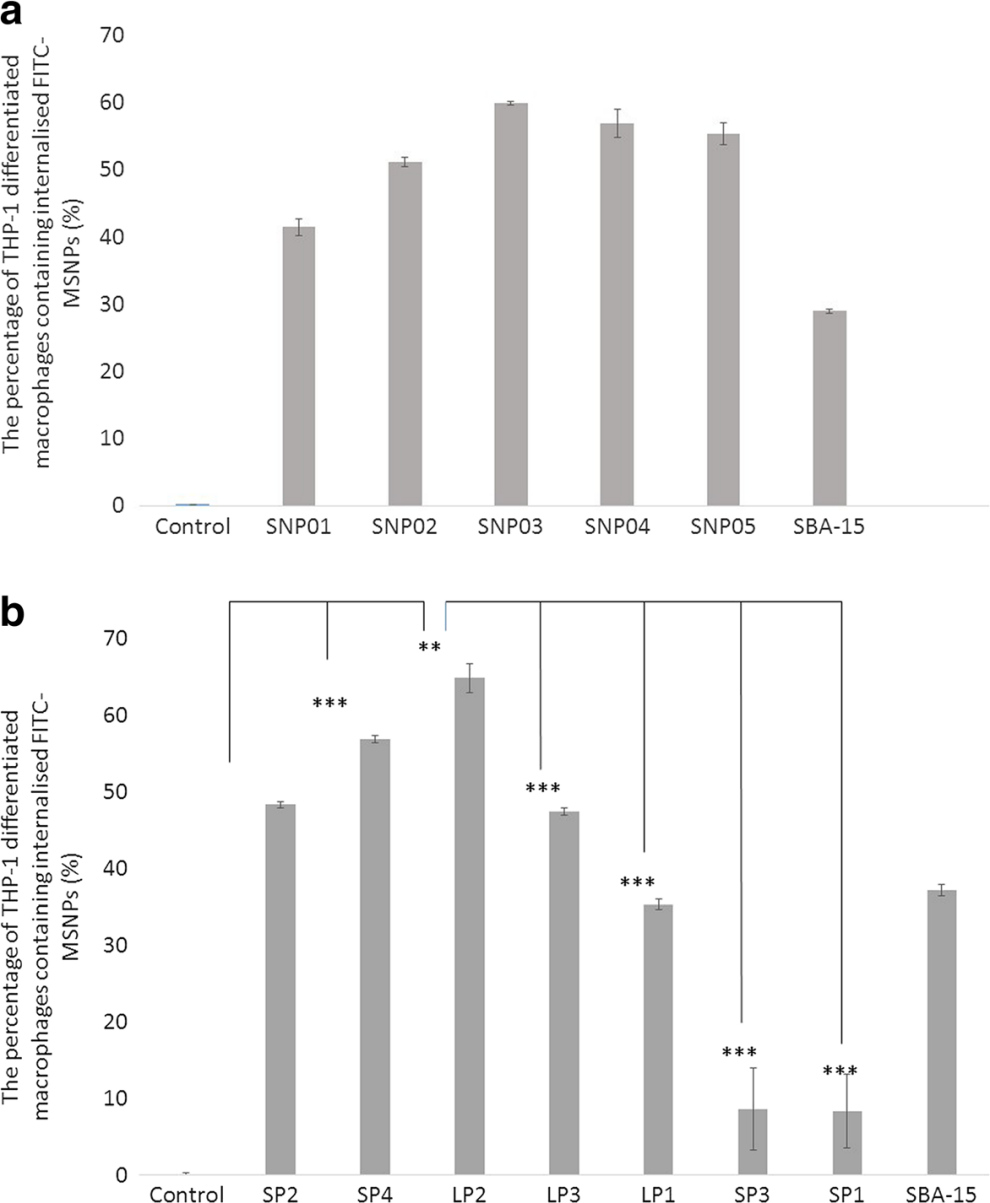
Unloaded FITC-labelled MSNPs were assessed by FACS for their ability to be internalized by macrophages. **a** Solid spherical nanoparticles [SNP]. **b** Mesoporous silica nanoparticle with small pores [SP] and large pores [LP]. SBA-15 was used in each data set for comparison. Data is presented as mean ± standard Deviation of triplicate samples and significance was tested using a one tailed t-test (*p < 0.05; **p < 0.01; ***p < 0.005)

The solid spherical particles were then tested for their ability to be internalized by macrophages. However, when using fluorescence as a marker on the particles, it is important to be able to differentiate between cell surface-associated particles and those which have been genuinely internalized. To control for this factor, all experiments were performed at room temperature, and then replicated at low temperature (Johnstone et al. 2001; Gottstein et al. 2013). Active transport of the nanoparticles into cells ceases under low temperature conditions, and therefore, any fluorescence seen would be due to surface attachment and so was subtracted. It can be seen that uptake increases from SNP01 to SNP03, i.e. in the order 113.25 nm, 275.78 nm and 377.63 nm (Fig. 2a). Subsequently, there is no significant increase in the amount of 588.00 nm internalized into the macrophages. Once the size reaches 847.25 nm, there is a significant decrease in the amount internalized compared to the peak uptake for 377.63 nm particles. This implies that there may be an optimal size for macrophage uptake of solid spherical silica nanoparticles peaking in the region of 377 nm. Although the individual SBA-15 is not as large as SNP05, the particles are rod-shaped and often severely aggregated, and therefore may not be taken up as readily.

While it is useful to look at the different uptakes of solid particles due to their identical morphology, they are unable to carry cargo. We therefore investigated the uptake of a series of mesoporous silica particles (Table 2). These particles have been previously characterized in terms of their capacity to carry antigen (Huang et al. submitted as Part 1 of 2). The size of the pores and the zeta potential is also reported in Huang et al. submitted as Part 1 of 2. It can clearly be seen that a similar pattern of uptake is seen with the MSNPs with respect to uptake and their external diameter. There is an increase in uptake from SP2 to LP2, i.e. 138 nm, 202.66 nm and 217.20 nm. There is a significant decrease in uptake between LP2 and LP3, LP1, SP3 and SP1, i.e. 217.20 nm and 217.75 nm, 462.33 nm, 496.96 nm and 1509.73 nm. Here, the rod-shaped SBA-15 particles were taken up to a similar extent to LP1, i.e. 612.75 nm and 462.33 nm, respectively. SP3 is only slightly larger than LP1, i.e. 462.33 nm compared to 496.93 nm, yet there is a very significant decrease in uptake. It is not clear why SP3 has a far lower uptake than SBA-15, although the synthesis method is slightly unusual in that it uses glycerol as the co-surfactant and co-solvent [23]. The zeta potential of the particles is all very similar and so not likely to cause significant differences [3]. SP1 has a very large external diameter (1509.73 nm), and this is most likely the reason why this particle has a very low uptake.

**Table 2:** The diameter of the mesoporous silica nanoparticles was assessed using CPS disc centrifuge. Data shows mean ± standard deviation

Particle name	Size
SP1	1509.73 ± 269.93
SP2	138.51 ± 26.29
SP3	469.93 ± 13.24
SP4	202.66 ± 19.65
LP1	462.33 ± 25.03
LP2	217.20 ± 22.66
LP3	217.75 ± 5.44

**Fig. 3:**
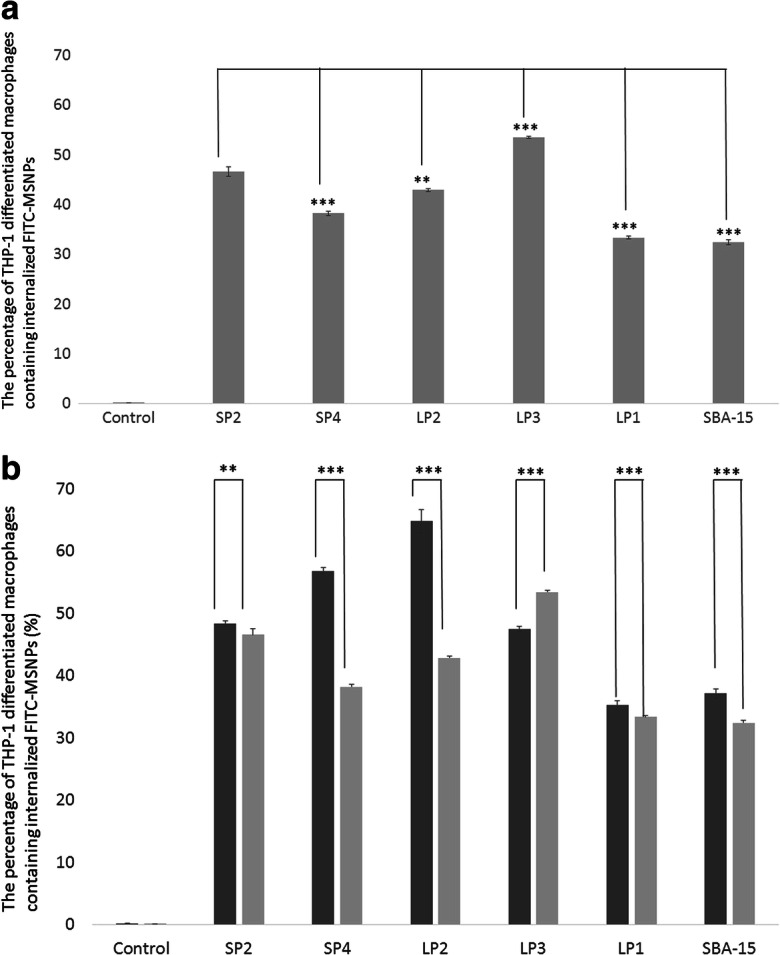
**a** Antigen loaded FITC-labelled MSNPs were assessed by FACS for their ability to be internalized by macrophages. **b** Comparison of loaded (light grey) and unloaded (dark grey) particles. Data is presented as mean ± standard deviation of triplicate samples and significance was tested using a one-tailed *t* test (*p < 0.05, **p < 0.01; ***p < 0.005)

The macrophage uptake experiments shown in Fig. 2b were for MSNPs without cargo. We subsequently tested whether the addition of antigen to the MSNPs would affect the uptake (Fig. 3). Due to their very poor uptake without cargo, SP3 and SP1 were omitted from this test (Huang et al. submitted as Part 1 of 2). The largest particle in this test, LP1 (462.33 nm) showed the lowest uptake (Fig. 3a), similar to results seen in Fig. 2b. The particles SP4 and LP2 showed the greatest changes in uptake after loading with diphtheria toxoid (Fig. 3b). SP4 uptake was significantly reduced (*p* < 0.005) from 56.8 ± 0.5 to 38.2 ± 0.4%, and LP2 uptake was significantly reduced (*p* < 0.005) from 64.8 ± 1.9 to 42.9 ± 0.3%. It is not clear why these particles would behave so differently since there is no observable difference in the particle surface charge, surface area or morphology that would explain why these two particles in particular had reduced uptake after loading.

We therefore looked at the hydrodynamic diameter of two of the MSNPs before and after loading see (Huang et al. An assessment of mesoporous silica nanoparticle architectures as antigen carriers). LP2 showed a very significant decrease in uptake into macrophages after loading. The size before loading was determined to be 217.20 ± 22.66 nm (Fig. 3a). After incubation with diphtheria toxoid at a ratio of 1:20 for 24 h at 4 °C, the size was 330.91 ± 4.86 nm, an increase of just over 50%. In terms of size the increase in size would place it nearer to LP1 (462.33 ± 25.03 nm). Unloaded LP1 showed considerably reduced uptake compared to LP2, and so, the size increase could explain the reduction in uptake (Fig. 3b). In contrast, LP3 which was seen to have a modest increase in macrophage uptake after loading only increased in hydrodynamic diameter from 217.75 ± 5.44 nm before loading to 270.61 ± 1.99 nm after loading.

**Fig. 4:**
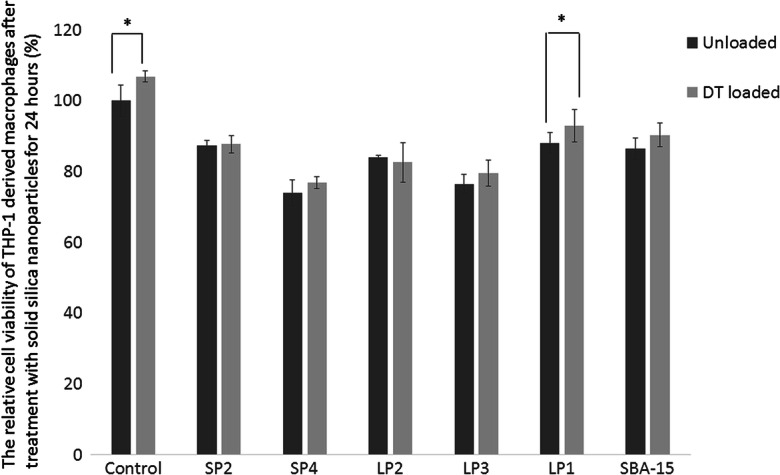
Relative viability of THP-1 derived macrophages 24 h after treatment with 10 μg/ mL unloaded FITC-MSNPs (black bars), or diphtheria toxoid loaded MSNPs (grey bars). Control indicates cells which have not been treated with any MSNPs. Data shows mean ± standard deviation (*n* = 3). DT diphtheria toxoid. (*p < 0.05; **p < 0.01; ***p < 0.005)

Once the ability of the macrophages to take up the various different particles had been determined, those which were taken up most readily were further examined for their effect on cell viability (Fig. 4). Both naked nanoparticles and those loaded with diphtheria toxoid were assessed. There was no difference seen between loaded and unloaded particles except for LP1 (*p* < 0.05). The LP1 particles showed the best biocompatibility as measured by survival of the macrophages 24 h after incubation with the particles, 88.1 ± 3.0% and 87.0 ± 4.6% for unloaded and loaded LP1, respectively. None of the particles, however, caused significant cell death with the lowest, DT-loaded SP4, showing a cell viability of 71.9% of the control.

Given the nanoparticle uptake by the cells (Figs. 2 and 3) and the relative viability of the cells (Fig. 4) after incubation with the particles, the performance of LP2 could be deemed the best, although with the exception of SP3 and SP1 which were poorly taken up by macrophages, all particles performed well. Interestingly, we have previously shown that SP3 and SP1 also showed the lowest loading efficiency for diphtheria toxoid (Huang et al. submitted as Part 1 of 2).

## Discussion

Macrophages are cells which form a part of the innate immune system and as such are able to engulf a wide variety of targets, including dead cells, dust, pollen and invading pathogens. Bacterial pathogens can differ greatly in their sizes, shapes and surface features, which can all have an effect on virulence (Cabeen and Jacobs-Wagner 2005). This can give indications to the way in which macrophages would interact with man-made materials such as the MSNPs designed to stimulate the immune system.

In order to perform their role in the immune system, macrophages engulf particles by the process of phagocytosis (Burke and Lewis 2002). Phagocytosis involves both structural rearrangements of the cytoskeleton and membrane, and also a complex network of signalling events. The process of phagocytosis is affected by the physical parameters of the particles being engulfed. These can be split into geometric aspects such as the size (Pratten and Lloyd 1986; Tabata and Ikada 1988a, 1988b; Koval et al. 1998), shape or aspect ratio of the particles (Lengerova et al. 1957; Champion and Mitragotri 2006; Gratton et al. 2008; Sharma et al. 2010; Lu et al. 2010; Champion and Mitragotri 2009), surface properties (Tabata and Ikada 1988a, 1988b; Faraasen et al. 2003; Roser et al. 1998; Gilberti et al. 2008; Ahsan et al. 2002) and mechanical aspects (Beningo and Wang 2002).

To study the uptake of the particles by macrophages, we used the immortalized cell line THP-1 and used PMA for differentiation of the cells. One problem associated with assessing uptake is whether it can be determined whether particles are attached to the surface of the cells, or have been truly internalized. Since the particles were labelled with FITC, they were to be observed via fluorescence. We therefore originally tried the methods which used the fact that trypan blue can quench green fluorescence from FITC (Avelar-Freitas et al. 2014). However, we found that the fluorescence was quenched from both external and internalized particles alike, so could not be used to differentiate uptake. We therefore explored the use of sodium azide, an inhibitor of phagocytosis, as a way to differentiate between particles which were phagocytosed compared to those which were merely surface bound. However, sodium azide has been shown to take up to 3 h of contact with cell culture before they have an effect on phagocytosis (Cifarelli et al. 1979) and can also inhibit oxidative phosphorylation. Since we were concerned that sodium azide could affect cell viability, this method was also not chosen. Consequently, we used low temperature to inhibit phagocytosis and subtracted any fluorescence which was detected after such treatment since this would be a result of surface-bound particles.

The highly cited silica particle SBA-15 was used as a comparison molecule in all our experiments. This is a rod-shaped particle with an aspect ratio of 3:1. Our results showed uptake in the region of 30% (Fig. 2a). The low uptake is not unexpected since aspect ratio is known to affect uptake, with very high aspect ratios completely inhibiting phagocytosis (Gratton et al. 2008).

Size has often been considered the most important factor for uptake, and the optimal size for macrophage internalization has been cited as between 1 and 3 μm (Pratten and Lloyd 1986; Koval et al. 1998; Tabata and Ikada 1988a, 1988b; Champion et al. 2008). This was thought to correspond to the size of a number of large bacteria (Kubitschek 1969). However, our largest particle (SP1; 1509.73 ± 269.93 nm) showed the lowest uptake, and the optimal uptake was for much smaller particles. Of course, as has been the case with regard to aspect ratio, the shape of the particles is also important. In 2006, Champion and Mitragotri first demonstrated that the local shape at the point of contact with the membrane governs whether phagocytosis will be initiated, whilst the size will determine whether the process is completed. In addition to size and shape, the mesoporosity of silica nanoparticles has also been indicated in their biological interactions (Asefa and Tao 2012). Compared to solid silica nanoparticles, the mesoporosity of the MSNPs results in lower rigidity which buffers the interaction with biological entities. This also results in lowered interfacial energy between the nanomaterials and the biological surfaces. In our study, the greatest uptake for solid particles was for the SNP03 (377 nm) particles, whereas for the mesoporous particles, LP2 (217 nm) had the greatest uptake. However, the uptake for the mesoporous LP2 was slightly higher than for the solid spherical nanoparticle SNP03. The size of the pores on the MSNPs did not seem to affect the uptake, which was related only to external diameter of the particles (Fig. 1).

Surface charge has also been shown to be an important parameter in the degree of uptake of particles. Thiele et al. (2001) found that positively charged particles showed much greater uptake. For non-metallic nanoparticles, there was also a correlation between positive charge and the ability to elicit an immune response. However, negatively charged non-metallic nanoparticles were associated with antigen-specific tolerance (Fromen et al. 2016). Despite these studies, there still needs to be further investigation into whether a generalized statement with regard to the charge of the nanoparticle and its influence on the immune response. Silica nanoparticles have an inherent negative charge due to silanol groups on the surface. The solid silica nanoparticles synthesized in this study show increasing negative ζ potential with increasing size (Table 1). It is therefore difficult to separate charge and size in determining which factor contributes to the degree of uptake. The behaviour of the particles with regard to uptake by the macrophages altered after the MSNPs were loaded with DT. The DT has an isoelectric point of 4.1 (Pappenheimer 1937) and therefore under low pH conditions, the DT would be positively charged. The silica is negatively charged and so the DT would be electrostatically attracted. This means that not only would loading with DT potentially change the hydrodynamic size, but also the apparent charge of the loaded particle.

In addition to the size, shape and charge of the particles, the material may also influence the interaction with the macrophages. The material from which a nanoparticle is made has a direct influence on the functions of the antigen-presenting cells (Marques et al. 2017). Gold nanoparticles are one of the most studied in vaccinology (see Marques et al. 2017 for summary), but other metals such as iron oxide and nickel have also been investigated. Polymers have also been tested and for example, poly (lactic-co-glycolic acid) (PLGA) delivery systems have shown adjuvant activity (Nicolete et al. 2011), with both humoral and cellular responses (O'Hagan and Singh 2003; Men et al. 1999; Carcaboso et al. 2004). Other materials that have been explored include emulsifying wax (Cui and Mumper 2002), lecithin-glyceryl monostearate (Sloat et al. 2010), albumin, gelatin (Zwiorek et al. 2008), collage, chitosan and alginate (Sloat et al. 2010). We have chosen to investigate silica since it has the benefit of being easily manipulated to form mesoporous particles which have a very high surface area. MSNPs have been shown to have intrinsic adjuvant activity and to potentiate antigen-specific T cell immune responses (Kupferschmidt et al. 2014; Mahony et al. 2013). Furthermore, silica is widely reported to be biocompatible, and the nanoparticles degrade (Coppi et al. 2004) to the relatively harmless silicic acid (Diaconu et al. 2010).

## Conclusions

More effective vaccines could benefit from improved, nanoparticulate delivery systems. Silica is a biocompatible material which has the potential to both carry antigen and act as an adjuvant. We investigated a range of sizes of silica nanoparticles to determine the effect on uptake by THP-1 macrophage-like cells. It was seen with both solid and mesoporous silica nanoparticles that smaller particles were less effectively taken up by macrophages. Optimal sizes appeared to be in the 200–400 nm range. We have previously demonstrated that antigen is stable on silica particles for at least 3 months (Huang et al. submitted as Part 1 of 2). MSNPs therefore present a viable alternative to current vaccine compositions.
